# The Multimodal Diagnostic Approach Necessary in Detecting Elusive Submucosal Laryngeal Cancer

**DOI:** 10.7759/cureus.44606

**Published:** 2023-09-03

**Authors:** Camilla S Reimer, Jayme R Dowdall

**Affiliations:** 1 Otolaryngology - Head and Neck Surgery, University of Nebraska Medical Center, Omaha, USA

**Keywords:** videostroboscopy, dysphonia workup, laryngeal imaging, dysphonia, squamous cell carcinoma, submucosal laryngeal cancer, glottic cancer, narrow band imaging

## Abstract

Submucosal laryngeal lesions have proven themselves to be a diagnostic challenge in the field of medicine, often presenting inconsistently between endoscopic visualization, various imaging modalities, and biopsy. The conflicting clinical picture can lead to a delay in definitive diagnosis and treatment. A variety of laryngeal imaging modalities exist that give a unique perspective of the tumor being evaluated and can be used in combination to clarify discrepancies in presentation.

This report describes the clinical course of an undiagnosed laryngeal squamous cell carcinoma (SCC) presenting with persistent dysphonia, dysphagia, and unilateral vocal fold immobility. A negative head and neck computerized tomography (CT) scan reduced the concern for cancer, so symptomatic treatment with vocal fold augmentation was performed. Augmentation curiously worsened the dysphonia and also may have delayed the process of definitive diagnosis. Upon presenting to the laryngology clinic, stroboscopy demonstrated no vibration of the affected vocal fold. Submucosal vascular irregularity was noted with narrow band imaging with a very subtle keratotic mucosal change raising suspicion for underlying malignancy. Despite two CT scans that failed to visualize the lesion initially, a biopsy revealed keratinizing SCC, which was subsequently staged as T3N0M0. The patient elected to receive radiation therapy alone given his medical comorbidities.

This case showcases the elusive ability submucosal laryngeal cancers have in diagnostic workups. Heavy reliance on any single diagnostic modality may be misleading, resulting in delayed diagnosis and treatment. An early, thorough, and multimodal approach that analyzes the cumulative results of a variety of diagnostic tools is essential in identifying and treating these elusive cancers in a timely manner.

## Introduction

Submucosal laryngeal lesions have proven themselves to be a diagnostic challenge in the field of medicine. Squamous cell carcinoma (SCC) of the submucosal larynx has a seemingly elusive ability to present inconsistently between endoscopic visualization, imaging, and biopsy. Reports of computed tomography (CT)-detected lesions with multiple negative biopsies and white light endoscopy attempts are not unusual [1,2]. Residing below the surface epithelium, these tumors often lack visual landmarks that guide direct biopsy, leading to an increased risk of inadvertent sampling of healthy adjacent tissue [3]. Negative biopsies lead to a substantial delay in diagnosis, with one study showing delays ranging from six weeks to nine months [2]. 

CT has been a diagnostic imaging modality of choice for the detection and staging of laryngeal cancers, shown to have a 74% sensitivity in malignancy detection [4,5]. Certain imaging findings that are more indicative of malignant lesions are sclerosis or lysis of laryngeal cartilage, multifocality, infiltrative quality, and associated adenopathy [3]. Magnetic resonance imaging (MRI) is also beneficial in identifying laryngeal pathologies and has been shown to have significantly higher sensitivity than CT in staging laryngeal cancers [6]. However, the potential for motion artifact and increased duration of patient discomfort often lead practitioners to use MRI as a complementary test to unclear CT scans. Other imaging modalities that have proven useful in the evaluation of laryngeal lesions include narrow-band imaging (NBI), autofluorescence, optical coherence tomography, contact endoscopy, and near-infrared fluorescence imaging [7]. Each modality provides a slightly different insight into the characteristics of the lesion in question. For example, NBI uses specific wavelengths of light in order to visualize and distinguish between the vascularity of the mucosal and submucosal layers, giving better insight into deeper levels of the laryngeal tissues [7]. 

The following case is a particularly unusual SCC that emphasizes the vitality of using a multimodal diagnostic workup for submucosal laryngeal cancers. 

## Case presentation

An 84-year-old male presented with symptoms of persistent dysphonia, dysphagia to thin liquids, and 10-15 pound weight loss that began concurrently with an upper respiratory infection, presumed to be COVID-19. Notably, he had a previous 20-year smoking history and endorsed current alcohol use. Flexible laryngoscopy revealed unilateral vocal fold immobility, but CT was unremarkable for any lesions. An outside otolaryngologist initiated a trial of steroids and omeprazole, but given the lack of symptom improvement, he underwent left vocal cord augmentation with injection. The patient’s shortness of breath with speech improved with the procedure, but his voice quality degraded significantly. His dysphagia remained unchanged. 

**Figure 1:**
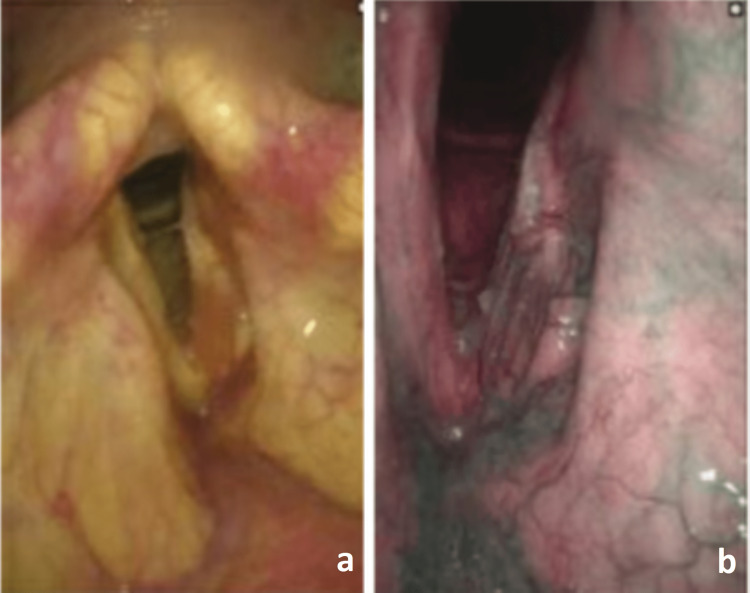
Images from flexible laryngoscopy reveal visualization of the vocal folds revealing erythema, leukoplakia, and contour changes in the left vocal fold (a). Increased vessel density of the anterior vocal fold can be noted on the narrowband image along with a subtle epithelial change just anterior to the vocal process (b). Of note, the area of subtle epithelial change does not appear to overlap with the increased density of blood vessels.

A laryngology referral was placed nine months after his initial visit. Laryngoscopy with white light revealed left true vocal fold immobility. No obvious contour changes were noted other than symmetric, age-related glottic atrophy. There was a slight blush of erythema to the left vocal fold, extending from the anterior to the vocal process to the anterior commissure (Figure 1a). Laryngovideostroboscopy revealed an erythematous, immobile left true vocal fold with no mucosal wave and an irregular, spindle-shaped closure pattern. A detailed evaluation of NBI revealed a subtle, diffuse, and slightly exophytic contouring of the left vocal process, extending about one-third the length of the posterior vocal fold. Some subtle epithelial changes and surface erythroleukoplakia were exhibited as well (Figure 1b). 

**Figure 2:**
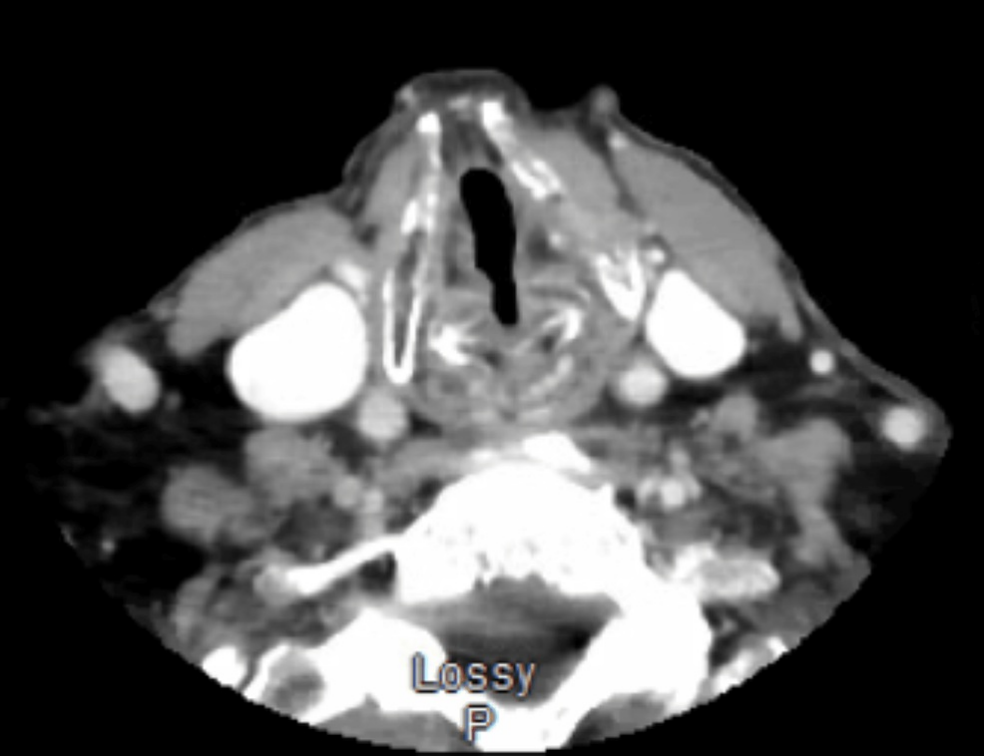
Repeat CT scan, which was interpreted by a radiologist as no evidence of vocal fold mass. CT, computed tomography.

Repeat head and neck CT did not initially visualize the lesion, nor any cervical lymphadenopathy (Figure 2). No additional findings were attributable to the vocal cord paralysis either. The differential at this time included a primary epithelial process due to frictional contact, neoplasm, and tissue reaction from the previous injection. 

Given the evidence of mucosal changes with visualization and NBI, the patient then underwent direct laryngoscopy with a biopsy of the left posterior true vocal fold and subsequent vocal cord injection. Pathology revealed an invasive keratinizing SCC, staged cT3, cN0, and cM0 (group stage 3). 

An institutional tumor board then re-evaluated the cumulative information available, including the endoscopic visualization techniques - white light, NBI, and videostroboscopy - CT imaging, direct laryngoscopy visualization, and biopsy pathology results. Given the vocal fold immobility and concern for underlying lesion characterized on endoscopy, the near imperceptibility of the lesion on CT scan, and the nests of neoplastic squamous cells with keratinization and stromal reaction on biopsy, the tumor board determined the given evidence to be sufficient for staging and treatment without further diagnostic imaging. 

After a discussion of surgical, medical, and radiation treatment options, the patient opted for laryngeal preservation with definitive radiation therapy. Given strong patient preference, advanced age with associated frailty, and negative nodal malignancy status, no adjunctive chemotherapeutics were utilized. 

## Discussion

The difficulty of the diagnostic course presented in this case emphasized the elusive ability that submucosal laryngeal cancers can have on various diagnostic modalities. The inconsistencies found between clinical presentation, endoscopic visualization, and initial imaging led to a delay in biopsy, definitive diagnosis, and appropriate treatment. 

In cases of unilateral vocal cord immobility, CT serves to detect focal lesions of the vocal cords and localize any definable impingement along the full length of the recurrent laryngeal nerve [6,8]. Primary laryngeal SCC is detected by CT with a sensitivity of 66% and specificity of about 74% [6]. Small or superficial tumors are less readily detected, but a lesion resulting in full fixation of the vocal cord, as in this case, would be expected to be appreciated in the scans [6]. Resultingly, this patient’s CT, which was negative for both primary laryngeal and extralaryngeal lesions, minimized his referring clinician’s concern for neoplasm. 

In pursuit of symptomatic treatment for his dysphonia, he underwent vocal cord augmentation with injection. While appropriate in cases of nonneoplastic and idiopathic vocal cord paralysis, this patient’s symptomatic treatment resulted in further clinical deterioration and delayed his referral for further diagnostic workup by nine months [9]. 

Upon further evaluation, the use of NBI allowed for the identification of a submucosal vascular abnormality that was not as readily appreciated with white light endoscopy alone, raising suspicion for an underlying neoplasm and providing guidance for subsequent tissue biopsy. NBI’s clinical utility is not isolated to this case, either, as studies have shown that the addition of NBI to the standard white light endoscopy improves the detection of cancers from 71% to 91% [7,10,11]. 

While no reports of SCC previously treated with vocal fold augmentation were readily identified, based on the results of a study showing a decreased sensitivity of tumor detection in both white light and white light/NBI combination endoscopies in patients with prior laryngeal surgeries, it can be speculated that the previous injection may have complicated tumor visualization further [11]. Regardless of previous operations, though, the combination of NBI and white light endoscopy proved superior in cancer detection [11]. 

In addition to NBI, a wide range of diagnostic tools exist to aid in the full evaluation of laryngeal lesions. These tools - including autofluorescence, MRI, optical coherence tomography, near-infrared fluorescence imaging, and contact endoscopy - have unique mechanisms of imaging that provide different perspectives of the tumor in question. The use of a combination of modalities to gain a full picture of the lesion in question is essential to avoid false negatives. 

Given the seemingly elusive nature that submucosal laryngeal cancers have in the diagnostic process, a thorough and multimodal approach to evaluation must be utilized to ensure proper and timely diagnosis and treatment. Reliance on the results of one or two modalities alone can lead to confusing clinical pictures and false assurance of a nonmalignant etiology of disease. Early utilization of a thorough and multimodal diagnostic process could have reduced the delay in diagnosis and proper treatment this patient experienced. 

## Conclusions

The diagnostic dilemma presented in this case emphasizes the necessity of a thorough, multimodal diagnostic approach in the detection of submucosal laryngeal SCC. The inconsistent visualization of this patient’s tumor on clinical exam and multiple imaging modalities in light of the pathologic biopsy results showcase the elusive ability submucosal laryngeal cancers can have in the diagnostic workup. While this is a unique case presentation, and other submucosal laryngeal SCC cases may present differently, this case emphasizes the vitally important knowledge that heavy reliance on any single modality may be misleading, resulting in a delay in definitive diagnosis and appropriate treatment. A thorough, multimodal approach early in the workup that analyzes the cumulative results of a variety of diagnostic tools - physical examination, direct biopsy, and multiple imaging modalities (CT scan, laryngovideostroboscopy, NBI, MRI, etc.) - is essential in definitively diagnosing and treating these elusive cancers in a timely manner. 
